# Sphingosine 1-Phosphate Receptor 2 Regulates the Migration, Proliferation, and Differentiation of Mesenchymal Stem Cells

**DOI:** 10.23937/2469-570x/1410014

**Published:** 2015-12-02

**Authors:** S Tucker Price, Thomas H Beckham, Joseph C Cheng, Ping Lu, Xiang Liu, James S Norris

**Affiliations:** 1 Department of Microbiology and Immunology, Medical University of South Carolina, Charleston, SC, USA; 2 Memorial Sloan Kettering Cancer Center, USA; 3 Department of Psychiatry, Medical University of South Carolina, Charleston, SC, USA

**Keywords:** Mesenchymal stem cells, Erk, S1PR2, Self renewal, Pluripotency factors

## Abstract

Mesenchymal stem cells (MSCs) are a multipotent cell population acquired most prominently from bone marrow with the capacity to differentiate into osteoblasts, chondrocytes, adipocytes, and others. MSCs demonstrate the capacity to home to sites of injury and contribute to tissue repair. Sphingosine 1-phosphate (S1P) is a biologically active sphingolipid impacting proliferation, apoptosis, inflammation, and angiogenesis with changes in S1P concentration providing significant implications for various disease conditions including cancer, diabetes, and cardiac disease. These functions are primarily mediated by interactions with 5 G-protein coupled S1P receptors (S1PR1-5). In this paper, we demonstrate that inhibition of S1PR2 results in increased MSC clonogenicity, migration, and proliferation; features dependent on Erk phosphorylation. Furthermore, decreased S1PR2 expression decreases the differentiation of MSCs into adipocytes and mature osteoblasts that may be the result of increased expression of MSC pluripotency factors including Nanog, Sox-9, and Oct-4. Inhibition of S1PR1 and S1PR3 in contrast does not impact MSC migration or Erk activation although increased proliferation is observed. In the study, we describe the essential role of S1PR2 in MSC differentiation pathways through modification of pluripotency factors. We propose a MAPK dependent mechanism through S1PR2 inhibition that promotes equally multipotent MSC proliferation.

## Introduction

Mesenchymal stem cells (MSCs) are critical components of bone marrow defined by their characteristic surface marker expression, plastic adherence, colony forming capacity, and pluripotency as established by the International Society for Cellular Therapy [1–4]. MSCs have critical physiological roles in the maintenance of the bone marrow stromal environment and the maintenance of hematopoietic stem cells. Peripherally, MSCs contribute to injury repair and regeneration at distant sites of injury and additionally have immunomodulatory functions [5]. The dual role of MSCs in bone marrow homeostasis and in remote tissue repair and signaling pathways that govern each function is still under investigation. Ongoing research seeks to better understand these functions and the mechanisms of MSC propagation, differentiation, and mobilization.

A combination of pro-differentiation and self-renewal factors dictate the proliferation rate and differentiation status of MSCs. Undifferentiated growth of MSCs can be promoted by a number of growth factors including fibroblast growth factor, platelet derived growth factor, and epidermal growth factor [6]. Activation of extracellular signal-regulated kinase (Erk) and Akt downstream of growth factor stimulation has been proposed as important signaling pathways promoting MSC self-renewal [7–11]. A number of different markers of a self-renewing MSC population have been identified including the transcription factors Oct-4, Sox-2, Nanog, and Rex-1 [11–14]. Depending on the pro-differentiation factors activated, MSCs can differentiate into various cell types including osteocytes, chondrocytes, adipocytes, myofibroblasts, fibroblasts, cardiomyocytes, and others [15,16].

Sphingosine 1-phosphate (S1P) is a metabolically active lipid involved in inflammatory, proliferative, and angiogenic processes in various cells types. S1P signaling can occur by both autocrine and paracrine mechanisms. S1P is synthesized from ceramide through the action of ceramidases and sphingosine kinases. The balance of S1P and ceramide has been shown to be critical in regulating growth and survival factors within a cell [17–20]. Most signaling properties attributed to S1P are through its stimulation of five specific G-protein coupled receptors that can signal through G_i_, G_q_, and G_12/13_ [17,21,22]. These receptors vary in their cellular expression with S1PR1-3 ubiquitously expressed and receptors 4 and 5 demonstrating more limited expression [23]. Each receptor has a characteristic signaling program involving all or a subset of these proteins. S1PR2 is unique among the S1P receptors in that it employs downstream signaling mediated by G_i_, G_q_, and G_12/13_ [24,25]. S1PR2, primarily through its strong G_12/13_ signaling has been identified as antagonistic to many of the canonical S1P functions. This has been best characterized with cell migration where it has been shown that S1PR2 in specific cell types can inhibit cell migration in vascular smooth muscle cells and cancer cell lines [26–30]. S1PR2 has additionally been shown to have essential functions in cell proliferation and survival, endothelial cell barrier function, mast cell activation, and auditory development [31,32].

S1P has been implicated in the promotion of MSC differentiation and mobilization, however the mechanisms of these effects require further investigation. S1P has been shown to promote differentiation of MSCs into smooth muscle cells through S1PR2 and S1PR3 signaling and into cardiomyocytes [33–35]. Within the hematopoietic compartment, S1P mediates cross talk between hematopoietic stem cells and MSCs to promote homeostasis [36,37]. Deficiencies in S1P degradation enzymes increase S1P concentrations in the blood as compared to the tissue or bone marrow [38–40]. Li et al. demonstrated that the elevated S1P generated by fibrotic liver injury was critical to the mobilization of green fluorescent protein (GFP) expressing bone marrow MSCs to injured tissues. Coordination between Erk, Rho Kinase, and Matrix Metalloproteinases has been implicated in mediating this effect [41,42]. Kong et al. recently proposed a role for S1P1 and S1P3 in the promotion of migration through an Erk dependent pathway with a simultaneously anti-migratory role for S1PR2 through the Rho kinase pathway [43]. S1P has been shown in other systems to serve as a chemoattractant to MSCs [15,37,44,45]. In this study, we uncover a new role for S1PR2 as a central signaling molecule in the differentiation of MSCs impacting MSC pluripotency factors.

## Materials and Methods

### Ethics statement

All cell preparation and all animal care were carried out with approval from the Medical University of South Carolina Institutional Animal Care and Use Committee under protocol #3108. MUSC has been fully accredited by the Association for Assessment and Accreditation of Laboratory Animal Care (AAALAC International) since 1987, with an unbroken record of compliance with regulatory inspections by the U.S. Department of Agriculture (USDA). MUSC’s Animal Welfare Assurance number is A3428-01.

### Cell culture and reagents

Murine MSCs were isolated from C57Bl/6 mice or GFP expression transgenic Bl/6 mice generously provided by Dr. Okabe [46–48]. S1PR2 KO mice were a gift from Dr. Richard Proia at the NIDDK on a FVB background with wild type FVB controls [49,50]. Whole bone marrow was harvested from the femurs of mice euthanized by CO_2_ inhalation. Additional digestion with Collagenase I (Sigma) was conducted prior to 70 µm cell filtration. Red blood cells were lysed and cells plated in Alpha Modified Eagles Medium (Invitrogen) containing 20% fetal bovine serum (Atlas Biologicals), L-glutamine (R&D), and pen-strep (Lonza). All cells were incubated in 5% CO_2_ at 37°C. After 3 days, non-adherent cells were discarded and adherent cells cultured for 4 passages. Cells were sorted on a BD FACS ARIA II cell sorter for CD45−, CD11b−, Sca-1+, CD73+, and CD105+ cells using antibodies obtained from BD Bioscience. Cells are used immediately following cell sorting. The Erk inhibitor U0126 was purchased from Cell Signaling. JTE013, dissolved in dimethylsulfoxide, and VPC, dissolved in ethanol, were purchased from Cayman Chemicals and FR180204 from Fisher Scientific. JTE013 is a selective S1PR2 antagonist and VPC is a competitive antagonist of S1PR1 and S1PR3 [51–55]. JTE013 was used at ranges up to 5 µM due to the high specificity for S1PR2 and low toxicity to the cells at this dose.

### Clonogenic survival assay

Clonogenic survival was assessed as previously described [56]. Passage 4 MSCs were plated in triplicate in 35 mm cell culture plates following treatment. Following 10–14 days in culture, cells were fixed in 3.7% formaldehyde and stained with 1% crystal violet. Colonies were counted when containing > 50 cells.

### Quantitative realtime PCR analysis

RNA was synthesized by RNEASY (Qiagen) from cultured cells according to manufacturer’s instructions and cDNA synthesis using the Biorad iScript cDNA synthesis kit. Real-time RT-PCR was performed with primers in Sybr Green Supermix (Biorad) was used for thermocycling reactions per manufacturers’ instructions. Cycling conditions were as follows: pre-incubation, 50°C for 10 minutes, 95°C for 3 minutes, followed by 25–35 cycles of denaturation at 95°C, 30 seconds; annealing/extension between 52 and 60°C, 45 seconds. Relative mRNA concentration was calculated as 2^-(CtTarget-CtCalibrator). All primers are shown in Supplemental table 1.

### Proliferation assays

MSCs were plated in 96 well plates at a concentration 500 cells/well. Treatment was delivered following cell attachment. Cell number was assessed using MTS assay (Fisher Scientific) with initial baseline evaluation immediately following treatment. Proliferation assays were discontinued if cells were determined to be unhealthy under microscopic observation or with medium pH changes. Alternatively cell number was assessed and quantified using the Essen Incucyte Zoom technology. The Incucyte Zoom takes serial high-resolution images of wells in a 96-well plate and calculates proliferation over the time course evaluated by using cell type specific parameters to identify cell borders and calculate percent confluence.

### Migration assays

Three experimental approaches to migration were taken. In the first, MSC migration was conducted using Incucyte Zoom evaluation of migration. Cells were plated to confluence in image locked 96-well plates coated with collagen I. Wound delivery was given using the 96 pin wound maker equipped with PFTE pin tips creating a 730–750 micrometer wound width. High definition phase contrast images were taken every fifteen minutes with cells identified using MSC specific parameters. Analysis was conducted using three types of analysis. The first is wound width, which determines the distance between each side of the scratch. The second is wound confluence that evaluates the area occupied by the cells as compared to initial wound area. The final analysis is relative wound density in which the spatial cell density of the wound area is compared to the cell density of unwounded areas. This parameter allows for correction based on drug toxicity or proliferation. Scratch assays were additionally performed manually in plates where 80–120,000 cells/well were plated to confluence in 24 well collagen coated plates. Cell scratches were initiated following cell attachment using 10–100 uL pipette tips and media changed to remove any detached cells immediately prior to treatment. Scratches were imaged every 6 hours until 24 hours using a Zeiss Axiovert 200 microscope examining at the same field of cells at each time point. Scratch distance and percent closure were quantified using Image J. A modified Boyden chamber assay was also performed to assess migration in which 30,000 were plated on cell inserts in a 24 well plate and monitored by fluorescence for migration as previously described [56].

### Differentiation assay

Differentiation was induced as directed in chamber slides by the R&D systems mesenchymal stem cell functional identification kit. Cells were plated to confluence at 30,000 cells per chamber in millicell ez slide 4 chamber slides. Following attachment cells were switched to induction media. Adipogenic supplementation included hydrocortisone, isobutylmethylxanthine, and indomethacin and osteogenic supplement included dexamethasone, ascorbatephosphate, proline, pyruvate and recombinant human TGFβ3. Induction media was changed every 2–3 days for 14 days for adipogenic media and up to 21 days for osteogenic media. Following completion of this time, cells were fixed, permeabilized with Triton x-100, and blocked. Osteogenic induction was evaluated by goat anti-mouse osteopontin and adipogenic induction was evaluated by goat anti-mouse FABP4. Secondary antibody stain to visualize included Alexafluor 555 donkey anti-goat for GFP+ cells or Alexafluor 488 donkey anti-goat for non-GFP transgenic cells. Cells were co-stained with nuclear stain To-pro-3. Imaging of stained cells was conducted using Zeiss LSM 510 META confocal microscope. A minimum of 10 images with 100 total cells were taken at 63× magnification and cell counted were obtained from each image. Differentiation was also assessed as described in the absence of induction media [57–59].

### Western blotting

Immunoblot analyses of cell lysates were performed as previously described using antibodies to detect p-Erk, total Erk, p-Akt (Ser 473), total Akt, Rac 1/2/3, RhoA, RhoB, Rho C, CD44, Nanog (Cell Signaling), glyceraldehyde-3-Phosphate dehydrogenase (GAPDH) (Santa Cruz Biotechnology sc-32233), and p38 (Gift of Dr. K. Kirkwood) [56].

### Statistical analysis

Unless otherwise indicated, data represent mean ± standard error for 3 independent experiments and were tested for statistical significance by one-sided student t-test. Comparison of multiple groups was conducted using ANOVA with Bonferonni posttest comparisons.

## Results

### Sphingosine 1-phosphate receptor expression in mesenchymal stem cells

**Figure 1:**
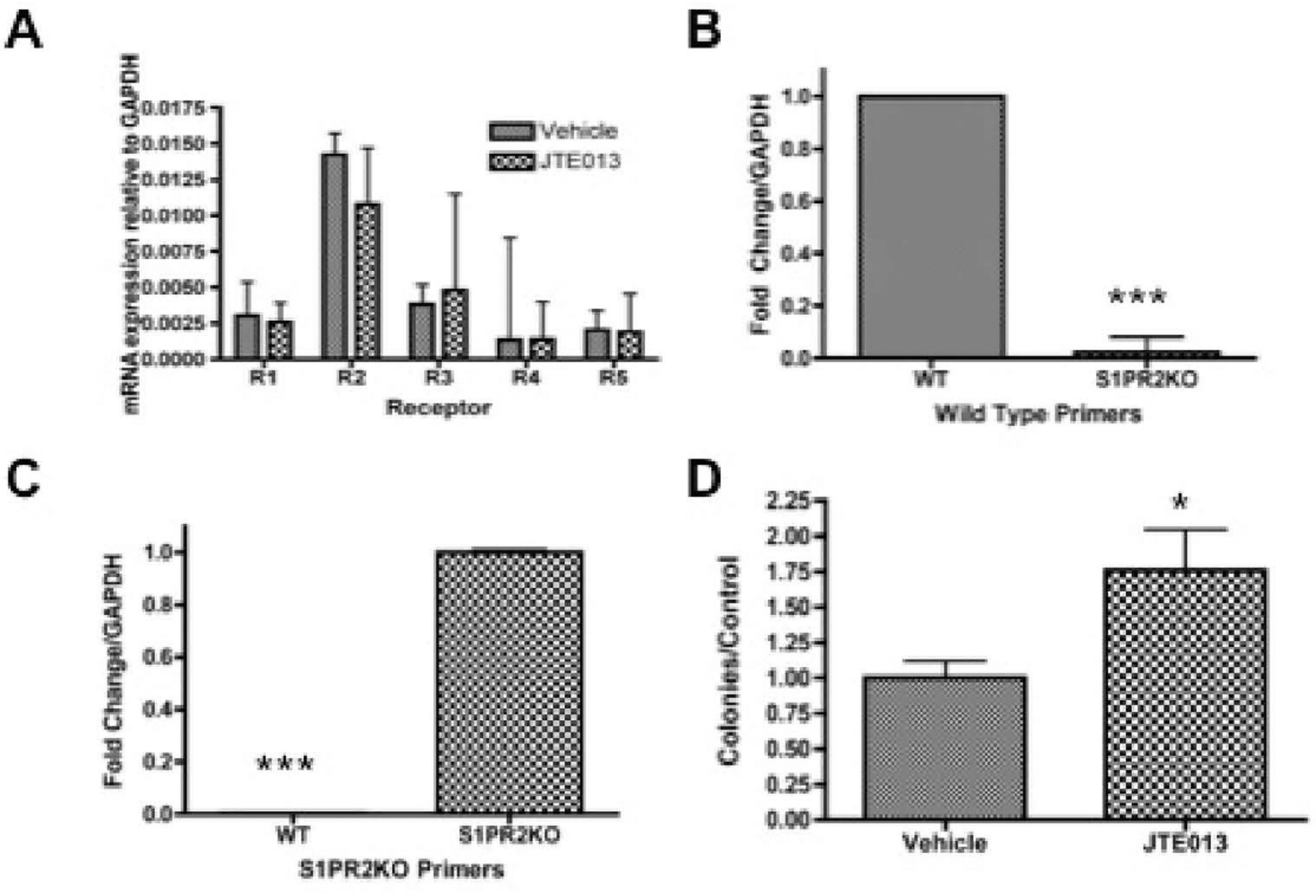
S1P receptor expression and clonogenicity- A) Relative mRNA concentration of MSCs treated with 1 µM JTE013 for 2 hours; B) Fold change for S1PR2 wild type primers in wild type and S1PR2 KO MSCs; C) Fold change for S1PR2 KO primers in wild type and S1PR2KO MSCs: D) Colony formation assay of Vehicle treated as compared to 1 µM JTE013 treated MSCS. Ten days after plating colonies were fixed in formaldehyde, stained with crystal violet and counted under a dissecting microscope. Data shown represents the mean ± sem for 4 independent experiments. ^*^ indicates p < 0.05 and ^***^ indicates p < 0.001 based on student t test analysis.

We first sought to assess the receptor expression of S1P receptors in murine MSCs to determine which receptors might be contributing to downstream S1P signaling in this cell population. To accomplish this, murine MSCs were assessed by quantitative real time PCR for S1P receptor expression. Relative mRNA concentration was calculated using GAPDH as a control as described in materials and methods. Consistent with their ubiquitous high receptor expression in various cell types, we demonstrate that S1PR1-5 mRNA is expressed in primary murine MSCs (Figure 1a). Both chemical and genetic manipulation of S1PR2 can be used to evaluate S1PR2 function. We therefore sought to address how both of these means of manipulation impacted transcriptional S1P receptor. Treatment with S1PR2 receptor inhibitor JTE013 did not significantly alter the receptor distribution (Figure 1a) [60]. Based on the mechanism of JTE013 as a competitive antagonist of S1PR2, it is not predicted to impact S1PR2 transcriptional expression. S1PR2 KO mice were demonstrated to have the appropriate knockdown with knockout mice expressing mRNA of the knockout insertion sequence instead of wild type mRNA (Figure 1b and Figure 1c). Genotyping further confirms the knockout genotype of these animals (Supplemental Figure 1).

Following our discovery that MSCs highly expressed S1PR2 messenger RNA, we sought to evaluate the functional importance of this receptor in MSCs and the potential impact on inhibition of receptor function. As clonogenic capacity and ongoing reproductive viability are critical factors in stem cell persistence and function, colony-forming capacity was evaluated. Clonogenic survival analysis indicated that treatment with the S1PR2 antagonist JTE013 significantly increased the clonogenicity of MSCs as compared to vehicle treated cells (Figure 1d). The observed increased clonogenicity does not appear to be the result of compensatory up-regulation of receptor expression, since JTE013 treatment does not change the receptor expression profile as shown in Figure 1a.

### S1PR2 inhibition promotes MSC proliferation and migration

**Figure 2:**
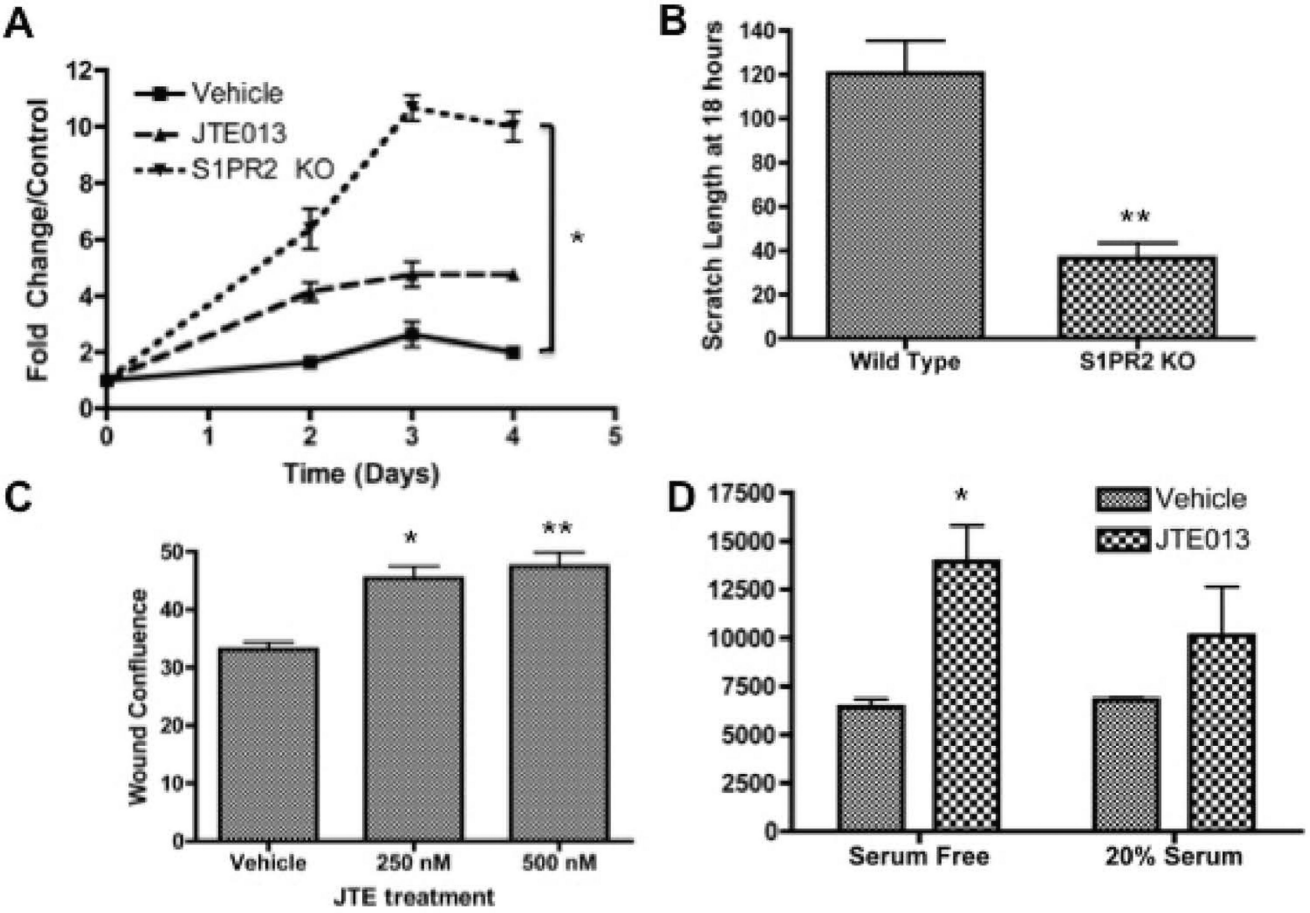
Inhibition of S1PR2 promotes increased cell proliferation and migration- A) MTS proliferation assay for MSCs from wild type, JTE treated, and S1PR2 KO mice normalized to the respective day 0 controls. Figure shown is a representative experiment of 3 total experiments; B) Scratch width of MSCs 18 hours after scratch for wild type and S1PR2 KO cells; C) Wound density analysis following scratch assay in MSCs 24 hours following treatment; D) Fluorescence 48 hours following modified boyden chamber migration assay with vehicle and 3 µM JTE013 treatment. ^*^ indicates p < 0.05 and ^**^ indicates p < 0.01 evaluated by a Student’s t test.

S1P has been identified as a critical mediator of cell survival and proliferation, although the S1PR2 can act antagonistically to this canonical activity of S1P. Based on changes in clonogenicity, we sought to determine the impact of S1PR2 inhibition on MSC proliferation. Both S1PR2 KO MSCs as compared to wild type MSCS and 3 µM JTE013 treated MSCS as compared to vehicle treated MSCs resulted in increased MSC proliferation as evaluated by MTS assay (Figure 2a). Analysis of the proliferation of MSCs 48 hours following JTE013 treatment indicated that increased proliferation appears to be dose dependent (Supplemental Figure 3). MSC migration is critical to the vascular, regenerative, and developmental roles of MSCs. The capacity of these cells to migrate toward an S1P gradient has been previously shown [3,36,42]. S1PR2 in other cells types has been characterized as inhibitory to cell migration through the antagonistic signaling of Rho and Rho kinases promoting cell migration and the anti-migratory signaling of Rac [26,45,61,62]. In MSC scratch wound assays, we demonstrate that S1PR2 KO MSCs migrate more quickly than their wild type counter parts. Figure 2b indicates that there is decreased scratch wound width in knockout cells as compared to wild type cells. Increased wound density 24 hours following JTE013 treatment at the indicated concentrations is shown in figure 2c as evaluated by Essen Zoom Incucyte software analysis. Representative images for figure 2b and figure 2c are shown in supplemental figure 2. We further find that JTE013 treatment additionally inhibits MSC migration when assessed by either wound density or percent migration (Supplemental Figure 4a and Figure 4b). When a modified Boyden chamber assay was conducted to evaluate MSC migration, MSCs treated with JTE013 demonstrated increased migration as compared with vehicle treated counterparts (Figure 2d). We therefore conclude that the loss of either expression or function of S1PR2 results in increased MSC migration.

### Erk phosphorylation mediates the proliferative and migratory changes in MSCs following S1PR2 inhibition

**Figure 3:**
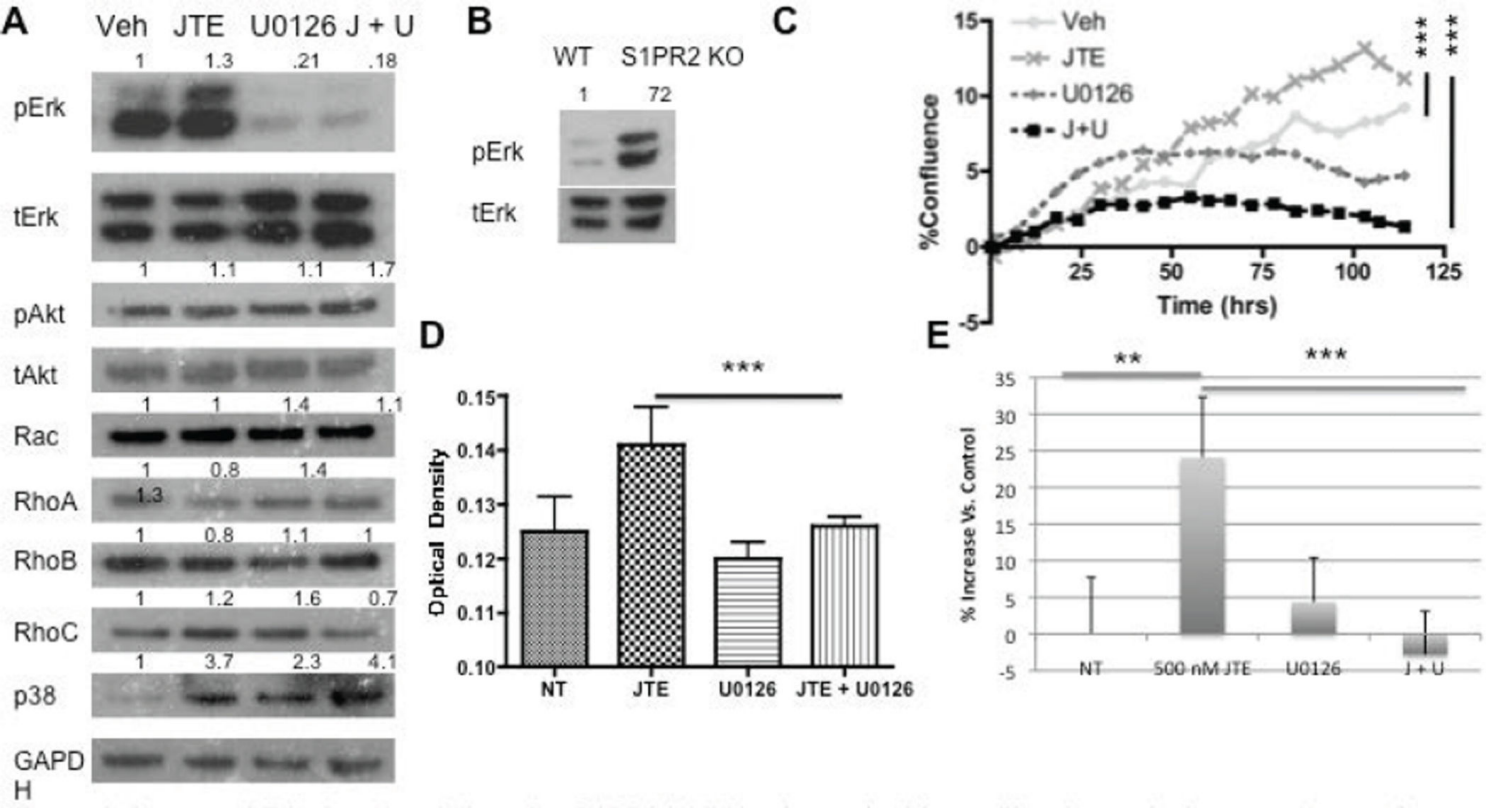
Increased Erk phosphorylation after S1PR2 inhibition is required for proliferation and migratory changes- A) Western blot analysis of protein activation or expression following 3 µM JTE013 and 1 µM U0126 treatment. A 30-minute U0126 treatment preceded the 30-minute JTE013 treatment. Densitometry reflects expression of the phosphorylated protein relative to total expression for pAkt and pErk or protein expression relative to GAPDH for the remainder; B) Western blot analysis of Erk activation in wild type and S1PR2 KO cells; C) Proliferation of MSCs with the indicated treatments evaluated by Essen biosciences analysis for confluence with p < 0.0001 by one-way ANOVA with bonferonni post test comparisons and similarly by MTS analysis; D) using the same concentrations at 48 hrs following treatment. P < 0.001 by one way ANOVA with bonferroni post test comparison; E) Scratch assay analysis of MSC migration normalized to the vehicle treated control for JTE and U0126 treated MSCs. ^*^ indicates p < 0.05, ^**^ indicates p < 0.01, and ^***^ indicates p < 0.001. Statistical evaluation by a Student’s T test unless otherwise indicated. Representative western blots are shown.

S1P has a number of well-characterized downstream signaling pathways following stimulation by S1P mediated by the activation of S1PR2 [24,51]. To better determine the signaling involvement of S1PR2 in proliferation and migration, we analyzed protein expression of these downstream pathways following S1PR2 inhibition As S1PR2 signals through G_i_, G_q_, and G_12/13_ we analyzed Ras/Erk, Rac, Rho, and p38 protein expression. Erk demonstrated increased phosphorylation following JTE013 treatment (Figure 3a). This increased phosphorylation was also observed in S1PR2 knockout MSCs as compared to wild type MSCs (Figure 3b). No changes in Akt phosphorylation or Rho and Rac protein expression were observed following JTE013 treatment indicating that they do not appear to be involved at a protein expression level in S1PR2 signaling in MSCs. A small increase in p38 expression was observed which requires further investigation into the significant of this increase. Treatment with UO126, an Erk inhibitor, decreased Erk phosphorylation both alone and in combination with 1 µM JTE013. Subsequent studies sought to address the role of Erk upregulation in S1PR2 mediated inhibition of MSC migration and proliferation. Inhibition of Erk in conjunction with S1PR2 inhibition results in abrogation of the previously observed increases in MSC proliferation by inhibition of S1PR2. This was demonstrated using MTS assay (Figure 3c) and by percent confluence of MSCs (Figure 3d). Similarly when Erk phosphorylation was inhibited in conjunction with S1PR2 inhibition, there was a significant decrease in the previously observed migration stimulated by inhibition of S1PR2. This is demonstrated in figure 3e using a scratch wound assay. To make our evaluation of Erk inhibition more comprehensive, similar experiments with proliferation and with migration were conducted using as second Erk inhibitor, Fr180204. Combined treatment with FR180204 and JTE013 yielded the same results as U0126 in proliferation and migration assays (Supplemental Figure 5a and Figure 5b). We therefore conclude that S1PR2 decreases Erk phosphorylation in MSCs to contribute to its inhibitory role in MSC migration and proliferation.

### S1PR2 promotes MSC differentiation into adipocytes and mature osteoblasts

**Figure 4:**
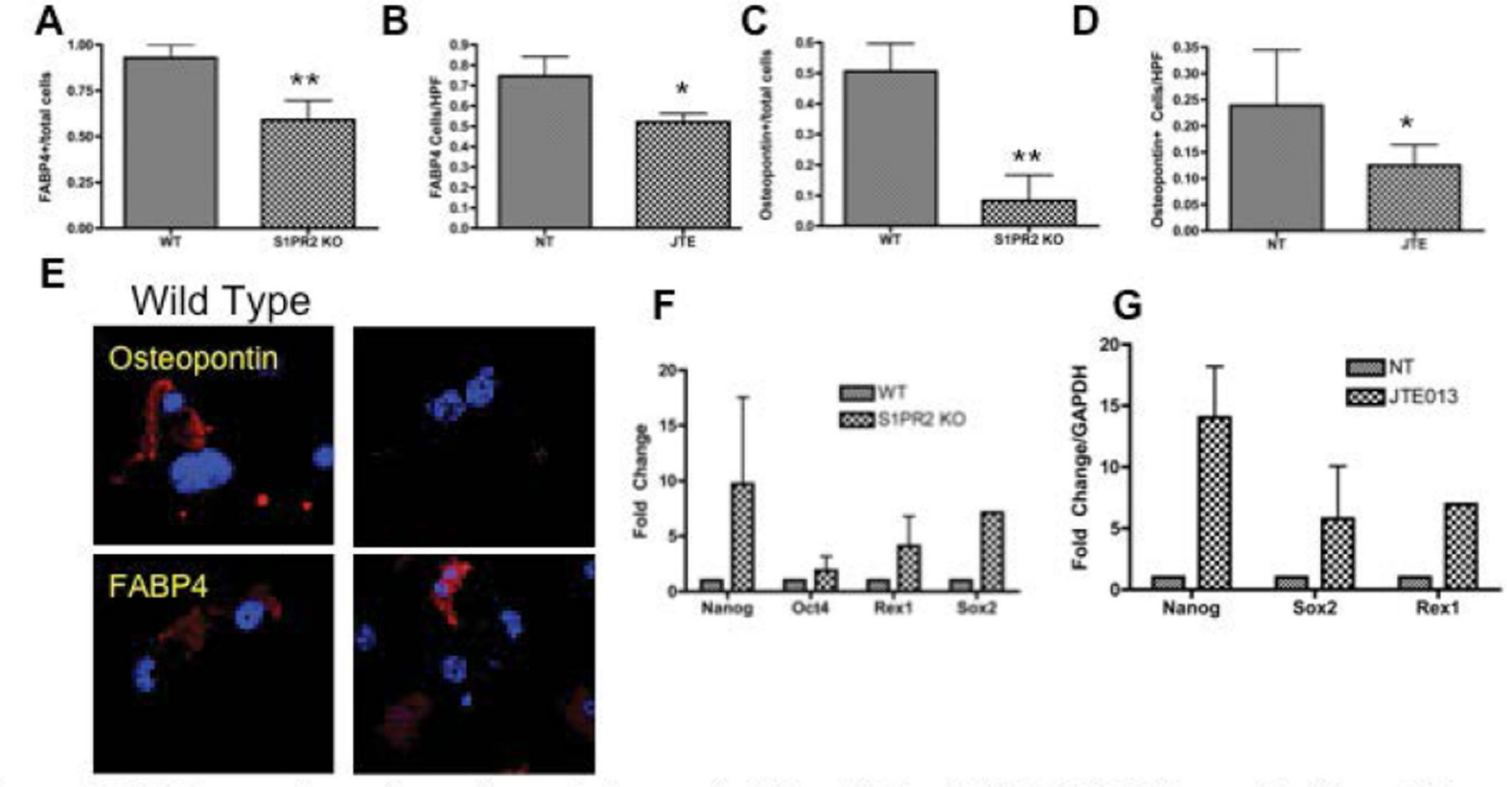
S1PR2 promotes adipogenic and osteogenic differentiation of MSCS- A-B Adipogenic differentiation of MSCs with S1PR2 KO MSCs and JTE013 treated MSCs following 21 day induction (A) and 21 day culture (B). S1PR2 osteogenic differentiation in induced S1PR2 KO cells (C) and 21 day cultured MSCs treated with JTE013 (D) E. Representative images of a HPF for S1PR2 KO and wild type cells for graphs quantified in A and C. F–G Quantitative realtime PCR analysis of MSCs in wild type and S1PR2 KO cells and 2 hour JTE013 treated MSCs (G). Results shown are a representative of results obtained in 3 independent experiments. HPF indicates a high-powered field used for analysis. * indicates p < 0.05 and ** indicates p<0.01 evaluated by a Student’s t test.

MSCs have the capacity to differentiate into both adipocytes and osteocytes. Adipogenic differentiation was evaluated by fatty acid binding protein 4 staining (FABP4) and osteogenic differentiation was evaluated by osteopontin staining [63,64]. S1PR2 KO cells display decreased FABP4 and osteopontin staining as compared to wild type cells following the respective adipogenic and osteogenic induction (Figure 4a, Figure 4b, Figure 4c and Figure 4d). Twenty-one day culture of 1µM JTE013 treated MSCs as compared to vehicle treated MSCs resulted in fewer cells staining for FABP4 and osteopontin. Representative images of the immunofluorescent staining for these differentiation markers are shown in figure 4e. Together, these two studies suggest that S1PR2 is critical to MSC differentiation into mature osteoblasts and adipocytes and that absence of receptor results in decreased differentiation into these lineages. Inhibition of S1PR2 results in increased expression of factors associated with increased pluripotency of MSCs (Figure 4f and Figure 4g) Transcriptional expression of pluripotency factors are increased with both JTE013 treatment and in S1PR2KO cells including Nanog, Oct-4, Rex-1, and Sox-2. S1PR2 is critical to MSC differentiation and inhibition of S1PR2 results in increased expression of factors critical to MSC propagation.

### Inhibition of S1PR1 and S1PR3 does not impact MSC migration and Erk activation status

**Figure 5:**
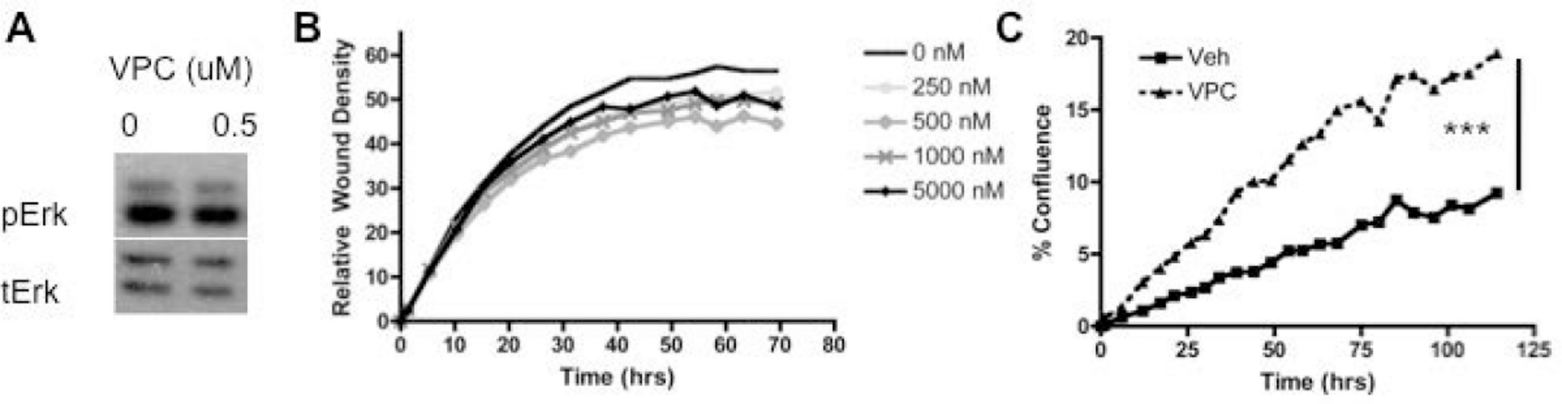
Inhibition of S1PR1 and S1PR3 does not impact MSC migration or Erk activation- A) Western blot analysis of VPC treated MSCs at the indicated concentrations for 30 min; B) Relative Wound Density analysis of scratch wound assay for VPC treated MSCs at the indicated concentrations; C) Proliferation analysis by cell confluence for VPC treated MSCs. ^***^ indicates p<0.001 as evaluated by a Student’s T test.

MSCs were treated with VPC23019 (VPC), a competitive antagonist of S1PR1 and S1PR3, and proliferation. 1 µM VPC treatment increased MSC proliferation when delivered throughout the duration of the proliferation assay (Figure 5c). Furthermore when migration analysis was conducted using increasing doses of VPC from 250 nM to 5 µM no significant differences in migration were observed in wound density and therefore migration (Figure 5b). Western blot analysis did not show any change in Erk phosphorylation following treatment with VPC (Figure 5a). Evaluation of the role of S1PR1 and S1PR3 using VPC treatment on MSC maintenance indicates that inhibitions of these receptors using VPC does not alter MSC migration or Erk phosphorylation.

## Discussion

MSCS are gaining increasing importance both in our understanding of their physiologic function in immunomodulation [65]. Increasing our understanding of how MSCs proliferate and differentiate in their reservoir areas including in the bone marrow will expand our knowledge on their role in physiologic function and maintenance. Our study addresses some of the properties that contribute to MSC expansion, proliferation and migration that contribute to their ability to perform these essential functions. We identify S1PR2 as a receptor that promotes cell differentiation and inhibits cell proliferation therefore acting antagonistically to the culture conditions required for *ex vivo* cell culture expansion of MSCs. Inhibition of S1PR2 promotes propagation of MSCs and enhances MSC proliferation. S1P is a critical lipid signaling molecule that promotes to cell proliferation and migration in a variety of cell types. Current research is starting to address receptor specific responses to S1P stimulation in cells of different origin. We have shown that inhibition of S1PR2 in bone marrow derived murine MSCs using genetic and pharmacological means results in increased MSC clonogenicity, proliferation, pluripotency and migration. This conclusion regarding the role of S1PR2 in murine bone marrow derived MSCs is in contrast to that generated by Quint et al. in their 2013 publication [36]. Our results differ from that of Quint et al. likely as a result of other nonspecific cell changes resulting from factors within the conditioned medium of the osteoclasts. Our findings that S1PR2 has a critical role in the inhibition of MSC migration through Erk phosphorylation are consistent with the findings of Kong et al. [43]. We additionally identify that this pathway is critical to S1PR2 proliferative changes.

S1PR2 has an inhibitory role in MSC proliferation and migration partially through its role in inhibiting Erk Phosphorylation. S1P stimulation canonically results in an enhancement of Erk phosphorylation through the Ras and Erk signaling pathway downstream of Gi, a target g protein of S1PR1-3 [66–68]. Inhibition of S1PR2 in MSCs, a cell type we have shown to have high S1PR2 expression relative to S1P1, results in increased Erk1 phosphorylation. The mechanism behind the interaction between Erk and S1PR2 could be the result of decreased inhibitory signaling through G12/G13 or through changes in the MAPK regulation as MKP-1 can also be upregulated following S1P stimulation [69]. Receptor compensation through increased Gi signaling in S1PR1 could also account for the increased Erk signaling in the condition of S1PR2 inhibition. Erk inhibition results in abrogation of the increases in proliferation and migration mediated by inhibition of S1PR2. Changes in protein expression and activation in the other common downstream signaling pathways downstream of S1PR2 are not impacted by genetic or chemical inhibition of S1PR2.

S1PR1 and S1PR3 do not appear to be involved in regulation of MSC migration as inhibition of these receptors does not impact MSC migration. However, recent reports have suggested that VPC23019 may have a bias toward inhibition of S1P receptor signaling through Gi over Gq [55]. G_q_ has been previously associated with S1PR3 related signaling through Rho activation that has been implicated in S1PR3 mediated migration [43]. Interestingly, recent work published by Hirata et al. demonstrate a role for S1PR3 in the expansion of aldehyde expressing human breast cancer cells in coordination with sphingosine kinase 1 further supporting a role for S1P in promoting stem cell self-renewal [70]. Further work in MSCs and other stem cell population using genetic tools and individual inhibition to characterize the role of S1PR1 and S1PR3 would be merited to further explore this relationship and to expand on current findings.

MSCs enable tissue repair and regeneration though a combination of factors including their immunomodulatory role, cytokine secretion and differentiation into cells required for the location [13,71–73]. MSC differentiation into osteocytes, adipocytes, smooth muscle cells, cardiomyocytes, fibroblasts, chondrocytes, neuronal cells, and many other cell types [74]. In this paper, we have shown that S1P is critical to MSC differentiation into osteocytes and adipocytes. In the absence of S1PR2 signaling MSCs, there is a significant reduction in MSC differentiation.

Previously published work has examined some of the signaling pathways and factors maintaining MSCs in an undifferentiated state [75]. In MSCs, increases in transcriptional and protein expression of Nanog, Oct-4, Sox-2, and Rex-1 direct a downstream signaling network promoting maintenance of a multipotent state although there remains some controversy over which pluripotency factors are involved [75–78]. Furthermore, increased CD44 expression has been demonstrated in MSCs in a less differentiated state [46]. The increases in these pluripotency factors following S1PR2 inhibition parallels the changes in differentiation observed in MSCs. The impact of S1PR2 inhibition on differentiation in both adipogenesis and osteogenesis combined with the impact on critical universal self-renewal markers places S1PR2 at a central role in MSC differentiation that would likely impact other cells types. These changes in differentiation may have significant implications in the capacity of MSCs to migrate to promote wound recovery at sites of injury and suggest an involvement of S1P and the S1P gradient in controlling MSC differentiation status. Although the effects of S1PR2 in promoting proliferation while simultaneously promoting an increased stem cell phenotype may initially seem contradictory, it is likely that the complexity of S1P signaling and receptor compensation or local signaling from growth factors may contribute to this dual effect. In this paper we have identified S1PR2 as a critical promoter of MSC differentiation through alteration transcriptional pluripotency factors.

## Supplementary Material

01
