# Supplementary material for: Sphingosine 1-Phosphate Receptor 2 Regulates the Migration, Proliferation, and Differentiation of Mesenchymal Stem Cells
Source: Int J Stem Cell Res Ther. Author manuscript; Available in PMC 2016 Jun 1. (PMC4888913; doi:10.23937/2469-570x/1410014)
Supplement: 01 [file NIHMS785016-supplement-01.pdf]

Supplemental Table 1: Quantitative real time pcr and genotyping primers

Gene	Forward Primer	Reverse Primer
S1PR1	CTCCACCGTGCTCCCGCTCTA	GGAGATGTTCTTGCGGAAGGTCAGG
S1PR2	GCGTGGTCACCATCTTCTCC	CGTCTGAGGACCAGCAACATC
S1PR3	CATCGCCTTCCTCATCAGTATCTTC	CACAATCACTACGGTCCGCA
S1PR4	GCACCTTGAGCATAACAGGA	CGGGGACAGACTGAGAGAGG
S1PR5	ACTGCTTAGGACGCCTGGAA	CCGCACCTGACAGTAAATCCTT
Oct4	ACACCTGGCTTCGGATTTCG	GGCGATGTGGCTGATCTGCT
Nanog	GGTTGAAGACTAGCAATGGTCTGA	TGCAATGGATGCTGGGATACTC
Sox2	GCACATGAACGGCTGGAGCAACG	TGCTGCGAGTAGGACATGCTGTAGG
GAPDH	CAATGACCCCTTCATTGACC	GATCTCGCTCCTGGAAGATG
Genotypying 1	GCAGTGACAAAAGCTGCCGAATGCTGATG	
Genotyping 2	AGATGGTGACCACGCAGAGCACGTAGTG	
Genotyping 3	TGACCGCTTCCTCGTGCTTTACGGTATCG	

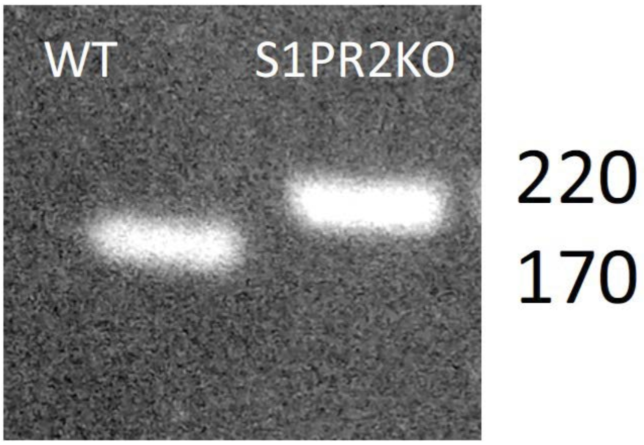

Supplemental Figure 1: Genotyping of S1PR2 KO animals A. PCR of DNA derived from the tail of wild type and knockout animals run with primers 1-3 shown in supplemental table 1.

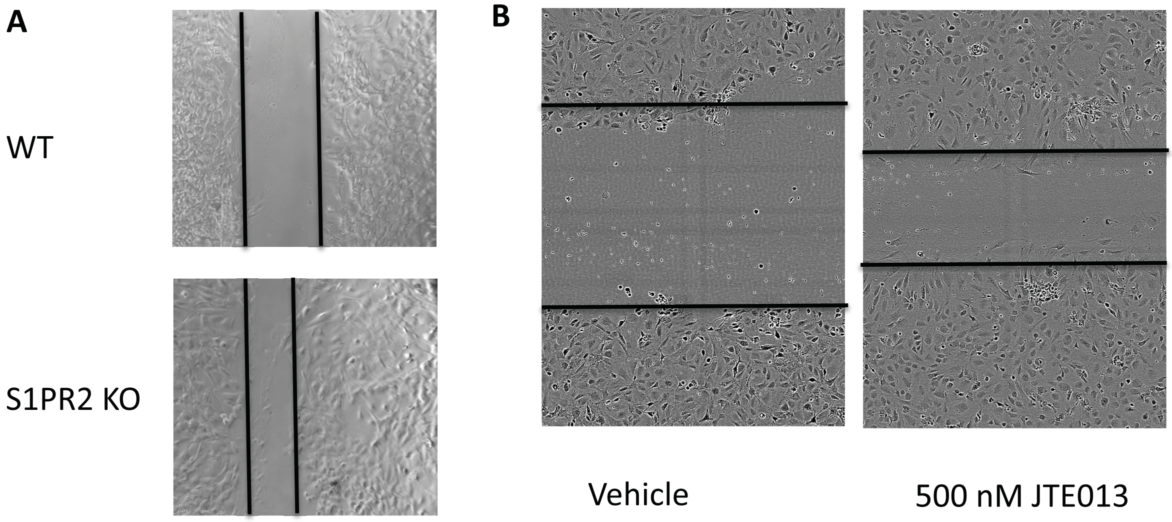

Supplemental Figure 2: Inhibition of S1PR2 promotes increased MSC migration. **A**) Wild type and S1PR2 KO MSCs were plated to confluence in 6 well plates for analysis of migration by scratch assay. Representative images from Figure 2b are shown; **B**) MSCs were treated with the indicated doses of JTE at the initiation of the scratch wound using Essen technology in a 96-well collagen coated plate. Representative images from Figure 2c are shown.

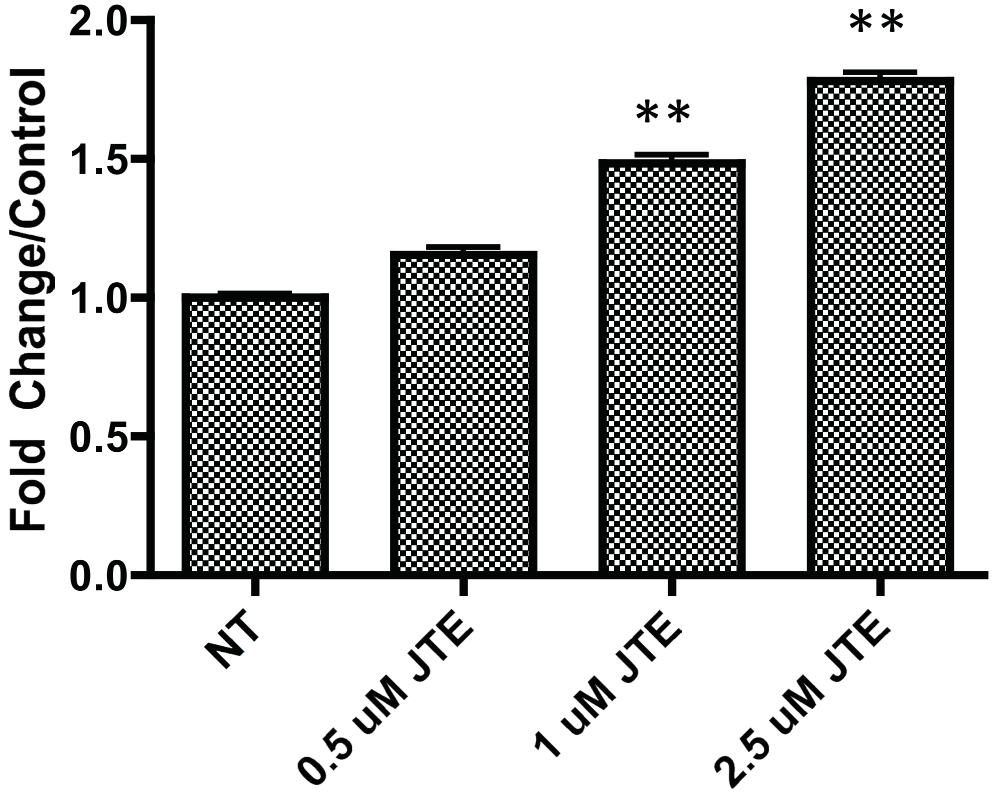

Supplemental Figure 3: JTE dose dependently increases MSC proliferation A. MTS evaluation of the proliferation of primary murine MSCs. Values represent the mean ± the standard deviation. Significance was assessed using a student's t-test for $p < 0.05$.

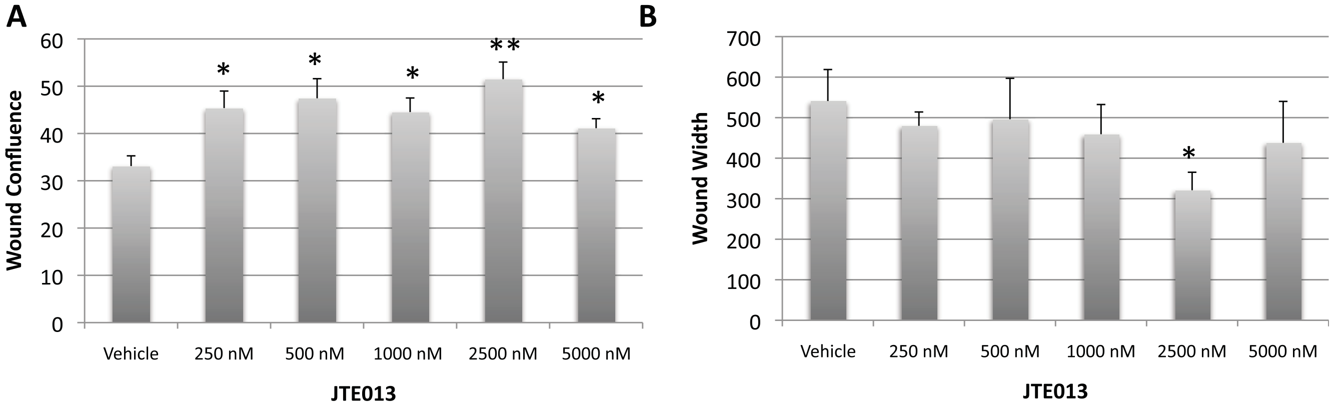

Supplemental Figure 4: JTE increases MSC proliferation A-B MSCs were treated with the indicated doses of JTE at the initiation of the scratch wound using Essen technology in a 96-well collagen coated plate. Wound confluence and width were evaluated by the Essen software based on a cell identification algorithm specific for the cells. Values represent the mean ± the standard deviation.

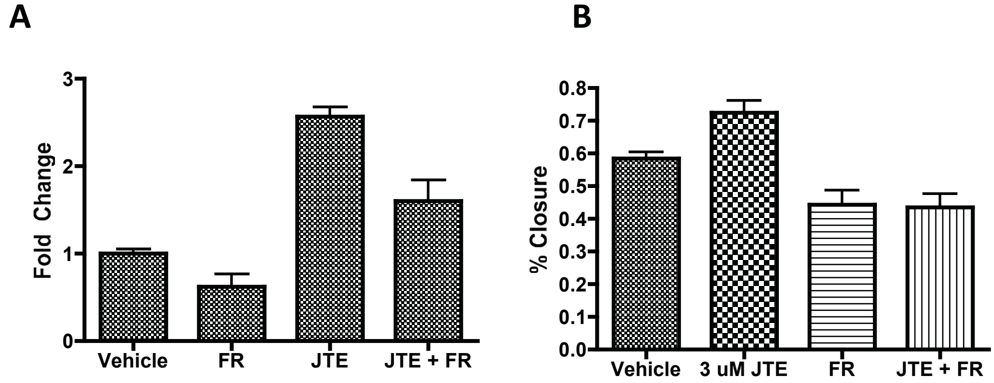

Supplemental Figure 5: Erk inhibitions abrogates JTE mediated increases in MSC proliferation **A**) MTS evaluation of MSC proliferation using primary murine MSCs treated with 1uM FR180204 delivered 30 minutes prior to JTE treatment; **B**) Wound closure of MSCs after 18 hours following scratch assay in primary murine MSCs. Groups are significantly different for A and B with $p < 0.0001$ by one-way ANOVA.
